# The impact of sarcopenia on clinical outcomes in men with metastatic castrate-resistant prostate cancer

**DOI:** 10.1371/journal.pone.0286381

**Published:** 2023-06-01

**Authors:** Efthymios Papadopoulos, Andy Kin On Wong, Sharon Hiu Ching Law, Lindsey Ze Jing Zhang, Henriette Breunis, Urban Emmenegger, Shabbir M. H. Alibhai

**Affiliations:** 1 Department of Medicine, University Health Network, Toronto, Ontario, Canada; 2 Joint Department of Medical Imaging, University Health Network, Toronto, Ontario, Canada; 3 Division of Epidemiology, Dalla Lana School of Public Health, University of Toronto, Toronto, Ontario, Canada; 4 Odette Cancer Centre, Sunnybrook Health Sciences Centre, Toronto, Ontario, Canada; 5 Department of Medicine and Institute of Health Policy, Management and Evaluation, University of Toronto, Toronto, Ontario, Canada; 6 Department of Supportive Care, Princess Margaret Cancer Centre, Toronto, Ontario, Canada; Kindai University Faculty of Medicine, JAPAN

## Abstract

**Introduction:**

Sarcopenia is common in men with metastatic castrate-resistant prostate cancer (mCRPC) and has been largely assessed opportunistically through computed-tomography (CT) scans, excluding measures of muscle function. Therefore, the impact of a comprehensive assessment of sarcopenia on clinical outcomes in men with mCRPC is poorly understood. The objectives of this study were to comprehensively assess sarcopenia through CT scans and measures of muscle function and examine its impact on severe treatment toxicity, time to first emergency room (ER) visit, disease progression, and overall mortality in men initiating chemotherapy or androgen receptor-targeted axis (ARAT) therapy for mCRPC.

**Methods:**

This was a secondary analysis of a prospective observational study of men with mCRPC at the Princess Margaret Cancer Centre between July 2015-May 2021. Participants were classified as sarcopenic if they had CT-based low muscle mass or low muscle density, a grip strength and gait speed score of <35.5kg and <0.8m/s, respectively, prior to treatment initiation. The impact of sarcopenia on severe treatment toxicity was assessed using multivariable logistic regression. Multivariable Cox regression models were used to determine the impact of sarcopenia on risk of visiting the ER, prostate-specific antigen progression, radiographic progression, and overall mortality.

**Results:**

A total of 110 men (mean age: 74.6) were included in the analysis. At baseline, 30 (27.3%) were classified as sarcopenic. Sarcopenia was a significant predictor of severe toxicity (aOR = 6.26, 95%CI = 1.17–33.58, P = 0.032) and ER visits (aHR = 4.41, 95%CI = 1.26–15.43, p = 0.020) in men initiating ARAT but not in men initiating chemotherapy. Sarcopenia was also a predictor of radiographic progression (aHR = 2.39, 95%CI = 1.06–5.36, p = 0.035) and overall mortality (aHR = 2.44, 95%CI = 1.17–5.08, p = 0.018) regardless of treatment type.

**Conclusions:**

Baseline sarcopenia predicts radiographic progression and overall mortality in men with mCRPC regardless of the type of treatment and may also predict severe treatment toxicity and ER visits in men initiating ARAT.

## Introduction

Sarcopenia is a geriatric syndrome that traditionally describes the progressive loss of muscle mass [1]. Since 2010, the definition of sarcopenia has been updated to include measures of muscle function, such as muscle strength and physical performance, in addition to muscle mass [2]. Several studies in oncology have opportunistically assessed sarcopenia through computed tomography (CT) or magnetic resonance imaging (MRI) scans, without including measures of muscle function, suggesting that sarcopenia is a prognosticator of anti-cancer drug toxicity [3] and overall mortality [4].

This opportunistic imaging-based method of assessing sarcopenia has gained attention in the prostate cancer (PC) setting, particularly, in men with metastatic castrate-resistance PC (mCRPC), a population that commonly exhibits profound impairments in muscle mass due to previous castration-inducing cancer treatments [5, 6] and advanced disease that is associated with increased muscle catabolic signaling [7].

Several studies in men with mCRPC have demonstrated that CT-based low skeletal muscle quantity/mass or muscle quality at the level of the 3^rd^ lumbar vertebra, notably also of the psoas muscle, are associated with a higher risk of grade I-II neutropenia during chemotherapy [8], disease progression [9, 10], and overall mortality [9, 11, 12]. Although these studies provided promising evidence of the role of sarcopenia in predicting clinical and disease outcomes in men with mCRPC, there was no consensus on the skeletal muscle measure (e.g., mass versus quality) that could best predict outcomes. More importantly, the definition of sarcopenia that was adopted by previous work in men with mCRPC [8–12] did not include measures of muscle function in addition to measures of skeletal muscle mass, whereas the latter are part of the updated definition of sarcopenia [2, 13, 14].

Muscle function for the purposes of evaluating sarcopenia includes assessment of muscle strength through grip strength and physical performance through gait speed or other tests, such as the Short Physical Performance Battery or Time-Up-and-Go Test [13]. Measures of muscle function are prognosticators of overall mortality [15], treatment modification [16], and complications [17] in older adults with cancer, and have been shown to better predict adverse outcomes than lean or muscle mass in non-oncology populations [18, 19].

Given that sarcopenia has been largely assessed opportunistically but not rigorously in oncology and particularly in men with mCRPC due to the lack of muscle function measures, the objectives of this study were as follows: a) to comprehensively assess sarcopenia using measures of muscle mass *and* function; and b) to examine the impact of sarcopenia on the time to the first emergency room (ER) visit, severe treatment toxicity, disease progression, and overall mortality in men with mCRPC.

## Methods

### Study population and design

This was a secondary analysis of a prospective multicentre observational study that was conducted at the Princess Margaret Cancer Centre, the Odette Cancer Centre, the Juravinski Cancer Centre and the Kingston Regional Cancer Centre in Ontario, Canada from July 2015 to April 2019 [20]. The objective of the original study was to assess treatment toxicity in men with mCRPC receiving chemotherapy or ARAT.

The inclusion criteria are published elsewhere [20]. In brief, the study included men ≥ 65 years old with histologically confirmed, radiologically documented metastatic PC with castrate testosterone levels who were initiating first chemotherapy treatment (docetaxel) or ARAT (enzalutamide or abiraterone). Participants who were starting ARAT were eligible to participate if they had not previously received chemotherapy. Participants were ineligible if they had a life expectancy of < 3 months, were not proficient in English, or had a neuropsychiatric disorder that interfered with participating in the study. Recruitment and completion of a baseline assessment occurred prior to treatment initiation for all participants. The baseline assessment involved collection of sociodemographic and disease characteristics, comorbidity information, and evaluation of grip strength and gait speed. Subsequently, participants were followed over time to provide information on potential treatment toxicities.

### Study procedures

For the purposes of this secondary analysis, an amendment was submitted to the Research Ethics Board to i) collect data on disease progression and overall mortality from the time of the baseline assessment until May 2021 and ii) retrieve a CT scan for each participant ≤ 6 months prior to treatment initiation. Given that access to CT scans from other sites was not feasible, this secondary analysis included only participants at the Princess Margaret Cancer Centre. Although participants had provided written informed consent form for the original study, the requirement for obtaining a separate consent for this secondary analysis was waived. The use of additional data for the present study was approved by the Ontario Cancer Research Ethics Board (Study ID: 1162) and Research Ethics Board at the University Health Network (Study ID: 15–9075.5).

### Outcomes

Study outcomes comprised severe treatment toxicity, time to the first ER visit, prostate-specific antigen (PSA) progression, radiographic progression, and overall mortality.

Severe treatment toxicity (i.e., grade 3+) was assessed during the original study every 3 weeks for participants on chemotherapy and every 1 to 3 months for participants on ARAT using the National Cancer Institute Common Terminology Criteria for Adverse Events version 4.0. All severe toxicities were recorded by the research coordinator based on interviews with patients and clinicians and chart review. The time to the first ER visit was assessed through medical records during the original study. PSA and radiographic progression were assessed through participants’ medical records from treatment initiation until treatment discontinuation or loss to follow up until May 2021. PSA and radiographic progression were determined using the Prostate Cancer Working Group 2 criteria [21], in line with previous studies [9, 10]. Overall mortality was assessed from treatment initiation until date of death until May 2021 and was confirmed through the Princess Margaret Hospital Cancer Registry supplemented by obituary searches for participants with no mortality date (i.e., “alive”) per the Princess Margaret Hospital Cancer Registry.

### Assessment and definition of sarcopenia

Sarcopenia was operationalized as a dichotomous variable (sarcopenic/non-sarcopenic) based on meeting the following two criteria: a) muscle weakness (<35.5kg) through grip strength and slowness (<0.8m/s) through gait speed per the Sarcopenia Definitions and Outcomes Consortium (SDOC) [19]; and b) low muscle mass or low muscle quality at the level of the 3^rd^ lumbar vertebra as described.

The research coordinator assessed both grip strength and gait speed at baseline, prior to treatment initiation. Grip strength was assessed three times in the dominant hand and the highest value was recorded. Gait speed was determined using the 4-meter walk and was expressed in m/s. An abdominal CT scan ≤ 6 months prior to treatment initiation was used for each participant to quantify muscle mass and muscle quality at the level of the 3^rd^ lumbar vertebra using 5 contiguous slices (0.82 x 0.82 x 2.5mm voxel size) centred symmetrically to the midpoint of L3, as determined using the 3D Slicer software [22]. Skeletal muscle mass at the L3 level is considered the gold standard for assessing sarcopenia through CT scans [23] as it correlates strongly with fat free mass [24] and is an independent prognosticator of adverse outcomes in adults with cancer [23, 25]. Muscle segmentation and quantification were performed using a histogram-based fully automated iterative threshold-seeking algorithm to optimize threshold selection for separating muscle from surrounding bone and fat. Location- and size-restricted organ-removal post-processing was performed since organs showed similar density values as muscle. Segmented areas smaller than 250 pixels were removed as suspected noise, with 3D connectivity between adjacent-slice muscle segmentations checked to ensure integrity of these noise-related island removal steps. This method was adapted from Wong et al [26] and applied within Jupyter Notebooks (Python 3.9). Manual corrections were performed on incorrect segmentations by exporting bone, muscle, and fat tags into SliceOmatic software (TomoVision, Montreal, Canada), with corrections re-imported back into Python for final metric computations. The automated algorithm was applied to all images to segment all muscle groups present within each slice. Manual corrections on Slice-O-Matic (TomoVision, Montreal, Canada) were completed by two members of the research team (EP & LZ) using a region-growing algorithm, restricting the Hounsfield unit (HU) range between -29 to +150 to further refine muscle mass and -190 to -30 for intra-muscular adipose tissue volumes, as suggested previously [24].

**Fig 1:**
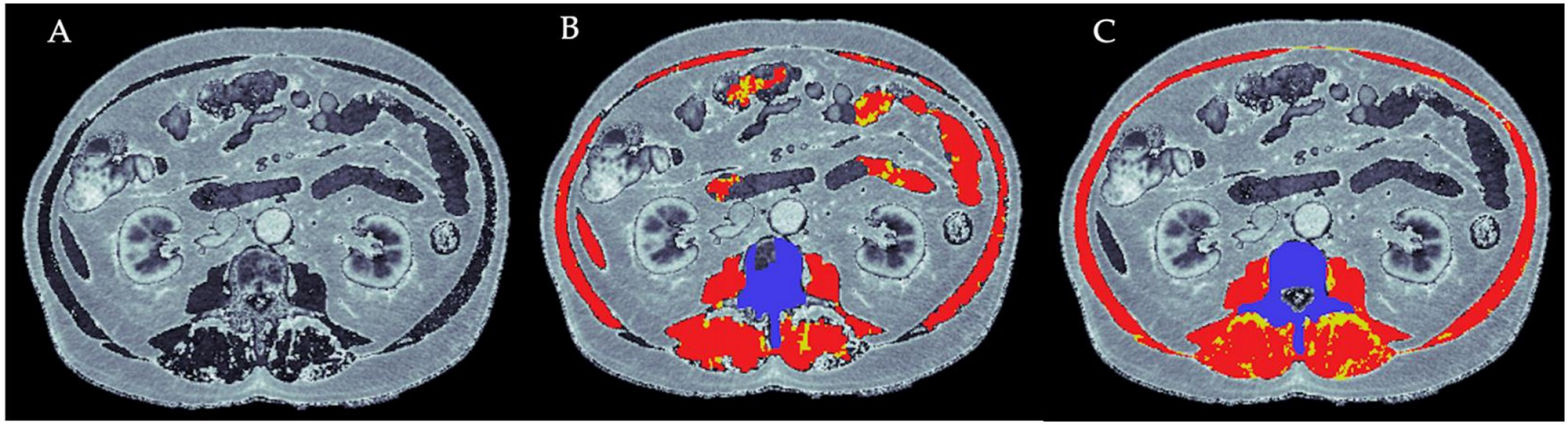
Assessment of total L3 level muscle mass and intramuscular fat. A) Raw L3-level CT central slice (0.82 x 0.82 x 2.5 mm) depicting muscle in dark grey (organs are also shown in dark grey) and fat in light grey. B) L3-level muscle, intramuscular fat, and bone segmentations achieved using the fully-automated algorithm. C) L3-level muscle, intramuscular fat, and bone segmentations after manual correction of automated outputs from B, using Slice-O-Matic software. Skeletal muscle segmentations are shown in red, fat in yellow, and bone in purple. Note: Processing of the image using the automated algorithm (B) resulted in mislabeling of some organs as muscle or fat. These inaccuracies were corrected using Slice-O-Matic (TomoVision, Montreal, Canada) (C).

Final corrected segmentations were used to quantify muscle cross-sectional area (in cm^2^) and muscle density in HUs (Fig 1). Data from the 5 slices for each participant were averaged, and the entire cross-sectional area was divided by the patient’s square of body height (in metres) to obtain the skeletal muscle index (SMI) that was used to quantify muscle mass/quantity. Muscle quality was expressed in HUs to represent the magnitude of fat infiltration in the skeletal muscle based on skeletal muscle density (SMD). Low muscle mass for patients with a body mass index (BMI) <25 kg/m^2^ was defined as having a SMI value of < 43cm^2^/m^2^, whereas for men with a BMI ≥25 kg/m^2^ a SMI value of <53 cm^2^/m^2^ was used [27]. Low muscle quality for men with a BMI <25 kg/m^2^ was defined as a HU value of <41, whereas for men with a BMI ≥25 kg/m^2^ a HU value of <33 was used [27].

### Statistical analysis

Continuous variables were summarized using means and standard deviations or the median and interquartile range as appropriate. Categorical variables were summarized using frequencies and proportions. Baseline characteristics between participants with and without sarcopenia were compared using independent sample t-tests and chi-square tests for continuous and categorical data, respectively.

Univariate and multivariable models were developed to study the associations between sarcopenia and study outcomes (treatment toxicity, time to first ER visit, PSA progression, radiographic progression, and overall mortality).

Logistic regression was used to determine the associations between sarcopenia and treatment toxicity. Kaplan-Meier plots and Cox regression models were used to assess the impact of sarcopenia on time to first ER visit, PSA progression, radiographic progression, and overall time to all-cause mortality. Covariates in multivariable analyses included variables that were not in the causal pathway for sarcopenia (e.g., instrumental activities of daily living or Vulnerable Elders Survey-13 [28]), had data missingness ≤10%, and were associated at p<0.05 with the outcome variable in the univariate analysis, apart from age that was included as a continuous variable in all multivariable models regardless of the univariate analyses. The proportional hazards assumption was assessed using visual techniques. An interaction term Sarcopenia by Treatment (i.e., ARAT vs. chemotherapy) was included in all multivariable models. A separate analysis by treatment type was performed when the interaction was marginally significant given our small sample. A natural log transformation was applied to covariates that were not normally distributed. Although ALP was missing in 10.9% of cases, it was included in the analysis given its prognostic value in PC-related outcomes [29]. Analysis of all-cause mortality included n = 99 participants given that n = 11 participants had re-enrolled in the study after transitioning from ARAT to chemotherapy. However, these 11 participants were included in the analysis of all other study outcomes and their sarcopenia status was reassessed prior to chemotherapy.

## Results

A total of 131 patients initiating chemotherapy or ARAT had participated in the original study, of whom 116 had an available CT scan ≤6 months prior to treatment initiation. Six (n = 6) participants were excluded due to missing grip strength and/or gait speed, leaving a total of 110 participants [mean age (SD): 74.6 (7.1) years] in the analysis, most of whom (57.3%) were initiating ARAT [abiraterone (16.4%); enzalutamide (40.9%)] (Table 1). Low grip strength was observed in the majority of the cohort (67.3%). Slowness was present in 32.7% of participants. Low SMI and low SMD were found in 82.7% and 78.2% of participants, respectively, whereas 27.3% of participants were classified as sarcopenic (Table 1). Chemotherapy was a more common treatment option for patients with sarcopenia. Apart from treatment type, no significant differences were found in clinical characteristics between patients with and without sarcopenia (Table 1). At baseline, the prevalence of hypertension was significantly higher in patients with sarcopenia, whereas congestive heart failure was more common in patients without sarcopenia (Table 1). Baseline differences in lactate dehydrogenase and hemoglobin between groups may indicate a more aggressive disease and inflammation in patients with sarcopenia. S1 Table in S1 File presents the number of events for all study outcomes. Specifically, 40.9% of participants visited the ER at least once during the study period, 38.2% experienced at least one grade 3+ toxicity, 60.0% had PSA progression, 45.5% had radiographic progression, while 64.6% of participants died from any cause during the study period.

**Table 1:** Baseline characteristics of study participants.

Characteristic	All participants n = 110	Sarcopenia^a^ n = 30	No sarcopenia n = 80	*p*	Missing n (%)
*Sociodemographic characteristics*					
Age (years), mean (SD)	74.6 (7.1)	75.7 (7.6)	74.1 (6.8)	0.30	0 (0)
BMI (kg/m^2^), mean (SD)	27.5 (4.7)	26.5 (4.9)	27.9 (4.6)	0.18	0 (0)
Race					0 (0)
Black	13 (11.8)	3 (10.0)	10 (12.5)	0.76	
South Asian	7 (6.4)	3 (10.0)	4 (5.0)		
Southeast Asian	8 (7.3)	3 (10.0)	5 (6.3)		
Unknown	1 (0.9)	0 (0)	1 (1.3)		
White	81 (73.6)	21 (70.0)	60 (75.0)		
Education, n (%)					0 (0)
Completed college/university or graduate degree	58 (52.7)	15 (50.0)	43 (53.8)	0.72	
*Clinical characteristics*					
Gleason score ≥ 8, n (%)	46 (48.9)	11 (45.8)	35 (50.0)	0.72	16 (14.5)
ADT duration (years), mean (SD)	6.2 (4.9)	6.4 (4.6)	6.1 (5.0)	0.73	0 (0)
Treatment type, n (%)				0.002	0 (0)
ARAT	63 (57.3)	10 (33.3)	53 (66.3)		
Chemotherapy	47 (42.7)	20 (66.7)	27 (33.8)		
Bone metastasis, n (%)	80 (72.7)	20 (66.7)	60 (75.0)	0.38	0 (0)
PSA most proximal to baseline, median (IQR), nmol/L	26.0 (8.9–76.1)	78.0 (76.0)	146.3 (806.9)	0.65	2 (1.8)
*Comorbidities*					
MoCA, mean (SD)	24.6 (3.6)	24.9 (3.5)	24.4 (3.7)	0.59	1 (0.9)
Dependence in one or more IADLs, n (%)	41 (37.3)	21 (70.0)	20 (25.3)	<0.001	1 (0.9)
VES-13 ≥3, n (%)	30 (27.3)	17 (56.7)	13 (16.3)	<0.001	0 (0)
Arthritis, n (%)	53 (48.2)	15 (50.0)	38 (47.5)	0.81	0 (0)
Congestive heart failure, n (%)	5 (4.5)	1 (1.3)	4 (13.3)	0.007	0 (0)
Diabetes, n (%)	22 (20.0)	8 (26.7)	14 (17.5)	0.28	0 (0)
Hyperlipidemia, n (%)	46 (41.8)	14 (46.7)	32 (40.0)	0.53	0 (0)
Hypertension, n (%)	60 (54.5)	21 (70.0)	39 (48.8)	0.046	0 (0)
Osteoporosis, n (%)	7 (6.4)	3 (10.0)	4 (5.0)	0.34	0 (0)
*Blood markers*					
Albumin (g/L), mean (SD) units	40.1 (3.5)	37.9 (3.6)	41.0 (3.1)	<0.001	15 (13.6)
Alkaline phosphatase (u/l), median, (IQR)	96.0 (74.0–143.3)	194.9 (184.2)	130.0 (150.1)	0.10	12 (10.9)
Lactate dehydrogenase (u/l), mean (SD)	260.9 (99.2)	308.14 (132.61)	240.5 (73.0)	0.016	17 (15.5)
Hemoglobin (g/L), mean (SD)	123.5 (15.9)	112.34 (17.0)	128.0 (12.9)	<0.001	10 (9.1)
Creatinine (IU/L), mean (SD)	99.7 (50.2)	111.1 (52.19)	94.9 (48.9)	0.14	10 (9.1)
*Sarcopenia-related measures*					
Grip strength (kg), mean (SD)	31.8 (7.8)	25.6 (4.7)	34.1 (7.4)	<0.001	0 (0)
Gait speed (m/s), mean (SD)	0.93 (0.2)	1.0 (0.2)	0.6 (0.1)	<0.001	0 (0)
Grip strength, n (%)					
<35.5kg	74 (67.3)	30 (100.0)	44 (55.0)	<0.001	0 (0)
Gait speed, n (%)					0 (0)
<0.8m/s	36 (32.7)	30 (100.0)	6 (7.5)	<0.001	
Days from CT scan to treatment initiation, mean (SD)	46.2 (41.2)	43.6 (37.8)	47.1 (42.6)	0.69	0 (0)
Skeletal muscle index, cm^2^/m^2^, mean (SD)	42.9 (7.4)	39.9 (6.5)	44.0 (7.3)	0.008	0 (0)
Muscle density (HU), mean (SD)	27.2 (9.8)	25.2 (9.3)	27.9 (9.9)	0.19	0 (0)
Low skeletal muscle index, n (%)	91 (82.7)	28 (93.3)	63 (78.8)	0.072	0 (0)
Low skeletal muscle density, n (%)	86 (78.2)	28 (93.3)	58 (72.5)	0.018	0 (0)

^a^Sarcopenia was defined as the presence of low muscle strength (grip strength <35.5kg), low gait speed (walking speed <0.8m/s), and low muscle quantity or quality.

ADT = androgen deprivation therapy; ARAT = androgen receptor-axis targeted therapy; BMI = body mass index; IADLs = instrumental activities of daily living; MoCA = Montreal cognitive assessment; PSA = prostate-specific antigen; VES-13 = Vulnerable Elders Survey-13

### Impact of sarcopenia on severe treatment toxicity

Table 2 lists the associations between sarcopenia and severe treatment toxicity. Sarcopenia was a significant predictor of severe treatment toxicity in the univariate model (OR = 3.27, 95%CI = 1.34–8.02, p = 0.009). The interaction of sarcopenia by treatment type did not reach statistical significance (p = 0.069). However, given that the interaction approached the level of significance and our small sample, a separate analysis was performed by treatment type (i.e., ARAT vs. chemotherapy) (S2 Table in S1 File). Sarcopenia was associated with a significantly higher risk of severe treatment toxicity in the ARAT (OR = 6.26, 95%CI = 1.17–33.58, p = 0.032) but not the chemotherapy group (OR = 0.87, 95%CI = 0.26–2.96, p = 0.82). No other significant predictors of severe treatment toxicity were observed in the multivariable analysis.

**Table 2:** Univariate and multivariable logistic regression of the impact of sarcopenia on severe treatment toxicity.

Variable	Univariate OR (95%CIs)	P	Multivariable OR (95%CIs) (n = 59) ARAT^a^	P	Multivariable OR (95%CIs) (n = 47) Chemotherapy^b^	P
Age per decade	1.29 (0.74–2.25)	0.36	1.02 (0.41–2.56)	0.96	1.51 (0.60–3.79)	0.37
Treatment type						
Chemotherapy	4.74 (2.05–10.93)	<0.001	N/A		N/A	
ARAT	ref.					
Sarcopenia						
Yes	3.27 (1.34–8.02)	0.009	6.26 (1.17–33.58)	**0.032**	0.87 (0.26–2.96)	0.82
No	ref.		ref.		ref.	
Anemia						
Yes	4.97 (1.55–15.75)	0.006	3.45 (0.63–18.92)	0.46	2.84 (0.39–20.37)	0.34
No	ref.		ref.		ref.	

ARAT = androgen receptor-axis targeted therapy; N/A = not applicable.

^a^Hosmer-Lemeshow test: 9.47, p = 0.30

C-stat: 0.77

^b^Hosmer-Lemeshow test: 2.73, p = 0.91

C-stat: 0.59

### Impact of sarcopenia on the time to first emergency room visit

**Fig 2:**
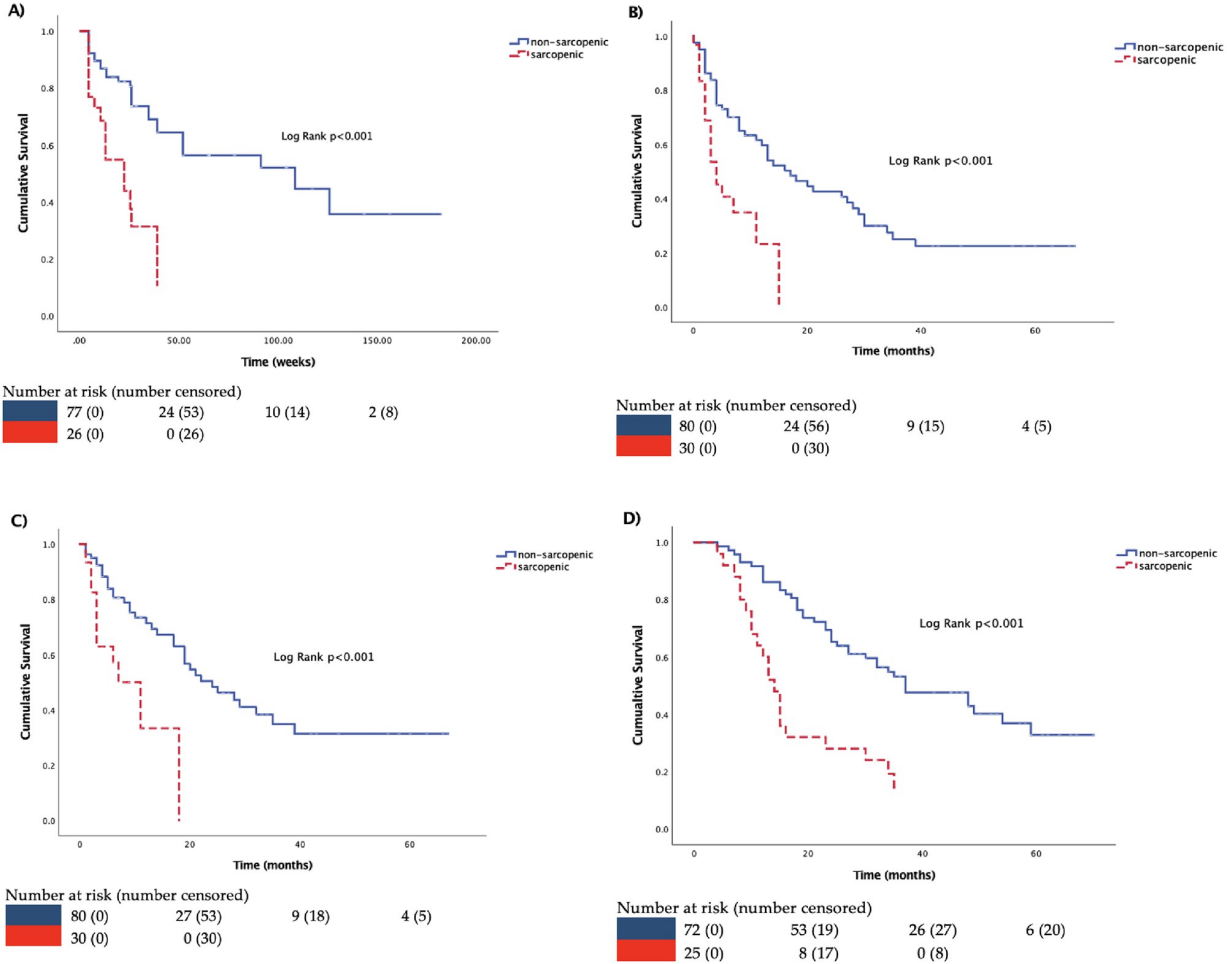
The associations between sarcopenia and A) time to the first emergency room visit, B) PSA progression, C) radiographic progression, and D) overall mortality. Survival was calculated using Kaplan-Meier plots and was compared using the log-rank test.

Table 3 lists the associations between sarcopenia and the time to the first ER visit during the study period. The median time to first visit to the ER was 6.6 weeks and 108.6 weeks for sarcopenic and non-sarcopenic participants, respectively (Fig 2a). Sarcopenia was an important predictor of visiting the ER (HR = 3.68, 95%CI = 1.92–7.05, p<0.001) in the univariate analysis. Separate multivariable analyses were performed by treatment given an interaction of marginal significance (p = 0.053) for sarcopenia by treatment (S3 Table in S1 File). Participants with sarcopenia initiating an ARAT had a significantly higher risk of visiting the ER (HR = 4.41, 95%CI = 1.26–15.43, p = 0.020) compared with non-sarcopenic participants. However, sarcopenia was not a predictor of ER visits in participants initiating chemotherapy (HR = 1.07, 95%CI = 0.45–2.52, p = 0.88).

**Table 3:** Univariate and multivariable Cox regression of the impact of sarcopenia on the time to the first emergency room visit.

Variable	Univariate HR (95%CIs)	P	Multivariable HR (95%CIs) (n = 49) ARAT	P	Multivariable HR (95%CIs) (n = 45) Chemotherapy	P
Age per decade	1.89 (1.24–2.89)	**0.003**	2.82 (1.18–6.70)	**0.019**	1.23 (0.63–2.39)	0.54
Treatment type						
Chemotherapy	9.37 (4.00–21.98)	**<0.001**	N/A		N/A	
ARAT	ref.					
Sarcopenia						
Yes	3.68 (1.92–7.05)	**<0.001**	4.41 (1.26–15.43)	**0.020**	1.07 (0.45–2.52)	0.88
No	ref.		ref.		ref.	
Anemia						
Yes	3.08 (1.21–7.83)	**0.018**	3.45 (0.41–29.20)	0.25	0.51 (0.13–1.98)	0.33
No	ref.		ref.		ref.	
Hypertension						
Yes	1.96 (1.06–3.66)	**0.033**	1.43 (0.47–4.40)	0.52	1.65 (0.67–4.08)	0.27
No	ref.		ref.		ref.	
Hemoglobin per 10g/L	0.74 (0.62–0.88)	**0.001**	1.06 (0.73–1.54)	0.76	0.82 (0.61–1.10)	0.18

ARAT = androgen receptor-axis targeted therapy; N/A = not applicable

### Impact of sarcopenia on PSA progression

Fig 2b shows that sarcopenic participants experienced PSA progression earlier compared with their non-sarcopenic counterparts. Specifically, the median time to PSA progression for sarcopenic and non-sarcopenic participants was 4 months and 17 months, respectively (p<0.001). Sarcopenia was significantly associated with PSA progression in the univariate analysis (HR = 2.77, 95%CI = 1.54–4.98, p<0.001) and although this association was also evident in the multivariable analysis (HR = 1.77, 95%CI = 0.91–3.48, p = 0.095), it was no longer significant likely due to the small sample size (Table 4). In the multivariable analysis, only initiating chemotherapy rather than ARAT was a significant predictor of PSA progression in this cohort (HR = 3.04, 95%CI = 1.45–6.35, p = 0.003) (Table 4). The interaction of sarcopenia by treatment type was not significant (p = 0.94).

**Table 4:** Univariate and multivariable Cox regression of the impact of sarcopenia on PSA progression.

Variable	Univariate HR (95%CIs)	P	Multivariable HR (95%CIs) (n = 97)	P
Age per decade	1.14 (0.79–1.62)	0.47	1.09 (0.74–1.63)	0.64
Treatment type				
Chemotherapy	3.78 (1.98–7.19)	**<0.001**	3.04 (1.45–6.35)	**0.003**
ARAT	ref.		ref.	
Sarcopenia				
Yes	2.77 (1.54–4.98)	**<0.001**	1.77 (0.91–3.48)	0.095
No	ref.		ref.	
ALP (log-transformed)	1.63 (1.09–2.42)	**0.016**	1.18 (0.78–1.81)	0.43
Hemoglobin per 10 g/L	0.82 (0.70–0.97)	**0.018**	0.95 (0.78–1.14)	0.56

ALP = alkaline phosphatase; ARAT = androgen receptor-axis targeted therapy

### Impact of sarcopenia on radiographic progression

Fig 2c demonstrates that participants with sarcopenia achieved radiographic progression earlier compared with non-sarcopenic participants. The median time to radiographic progression for sarcopenic and non-sarcopenic participants was 11 months and 24 months, respectively (p<0.001). Sarcopenia was a significant prognosticator of radiographic progression in the multivariable analysis (HR = 2.39, 95%CI = 1.06–5.36, p = 0.035) (Table 5). Additional predictors of radiographic progression included initiating chemotherapy versus ARAT (HR = 4.55, 95%CI = 1.68–12.35, p = 0.003) and higher creatinine levels (HR = 3.78, 95%CI = 1.61–8.86, p = 0.002) (Table 5). The interaction of sarcopenia by treatment type was not significant (p = 0.73).

**Table 5:** Univariate and multivariable Cox regression of the impact of sarcopenia on radiographic progression.

Variable	Univariate HR (95%CIs)	P	Multivariable HR (95%CIs) (n = 96)	P
Age per decade	1.56 (1.04–2.33)	**0.031**	1.24 (0.75–2.04)	0.39
Treatment type				
Chemotherapy	3.39 (1.55–7.45)	**0.002**	4.55 (1.68–12.35)	**0.003**
ARAT	ref.		ref.	
Sarcopenia				
Yes	3.27 (1.62–6.61)	**<0.001**	2.39 (1.06–5.36)	**0.035**
No	ref.		ref.	
ALP (log-transformed)	1.66 (1.04–2.64)	**0.032**	1.31 (0.81–2.13)	0.26
Hemoglobin per 10 g/L	0.80 (0.67–0.98)	**0.028**	1.05 (0.83–1.33)	0.69
Creatinine (log-transformed)	3.16 (1.59–6.27)	**<0.001**	3.78 (1.61–8.86)	**0.002**

ALP = alkaline phosphatase; ARAT = androgen receptor-axis targeted therapy

### Impact of sarcopenia on overall mortality

Participants without sarcopenia had a prolonged survival than participants with sarcopenia. The median time to overall mortality for sarcopenic and non-sarcopenic participants was 14 months and 37 months, respectively (p<0.001) (Fig 2d). Sarcopenia was associated with a higher risk of overall mortality in the univariate (HR = 2.99, 95%CI = 1.73–5.18, p<0.001) which was preserved in the multivariable (HR = 2.44, 95%CI = 1.17–5.08, p = 0.018) analysis (Table 6). Age per decade (HR = 1.97, 95%CI = 1.29–3.02, p = 0.002) and initiating chemotherapy versus ARAT (HR = 3.15, 95%CI = 1.77–5.61, p<0.001) were additional predictors of overall mortality in the multivariable analysis (Table 6). The interaction of sarcopenia by treatment type was not significant (p = 0.21).

**Table 6:** Univariate and multivariable Cox regression of the impact of sarcopenia on overall mortality.

Variable	Univariate HR (95%CIs)	P	Multivariable HR (95%CIs) (n = 86)	P
Age per decade	1.69 (1.16–2.48)	**0.006**	1.97 (1.29–3.02)	**0.002**
Treatment type				
Chemotherapy	3.45 (2.08–5.73)	**<0.001**	3.15 (1.77–5.61)	**<0.001**
ARAT	ref.		ref.	
Sarcopenia				
Yes	2.99 (1.73–5.18)	**<0.001**	2.44 (1.17–5.08)	**0.018**
No	ref.		ref.	
Congestive heart failure				
Yes	3.23 (1.27–8.17)	**0.013**	1.29 (0.46–3.61)	0.62
No	ref.		ref.	
ALP (log-transformed)	1.59 (1.04–2.44)	**0.031**	1.18 (0.79–1.77)	0.43
Hemoglobin per 10 g/L	0.76 (0.65–0.89)	**<0.001**	0.94 (0.76–1.16)	0.54

ALP = alkaline phosphatase; ARAT = androgen receptor-axis targeted therapy

## Discussion

The aim of this secondary analysis of a prospective observational study [20] was to comprehensively assess sarcopenia in men with mCRPC initiating chemotherapy or an ARAT and examine its impact on clinically relevant outcomes.

Previous studies in men with PC have assessed sarcopenia based on CT scans using the entire cross-sectional skeletal muscle area at the level of the 3^rd^ lumbar vertebra, or of the psoas muscle [8–12, 30]. Although this opportunistic method provides accurate estimates of muscle mass and density, it omits other important elements of sarcopenia, such as measures of muscle function that have been included in the updated definition per expert study groups on sarcopenia [2, 14, 19, 31]. Exclusion of muscle function measures from the definition of sarcopenia may substantially alter the proportion of participants who are classified as sarcopenic. For example, in line with previous studies in PC [10, 30], CT-based sarcopenia was found in the present study in 82.7% of patients. However, inclusion of measures of muscle function decreased the proportion of patients with sarcopenia to 27.3%. Additionally, sarcopenia in previous studies was assessed based on CT-derived muscle mass indicated by low SMI while muscle quality was examined separately. Muscle quality, however, appears to influence muscle function [32] and is included in the definition of sarcopenia according to the European Working Group on Sarcopenia in Older People [2]. Therefore, in our study, participants who exhibited low muscle strength and slowness in addition to low muscle mass or low muscle quality were classified as sarcopenic.

Meta-analytic evidence of 31 studies in patients with cancer demonstrates that CT-based low muscle mass is a predictor of more severe toxicity than patients with normal muscle mass [3]. Measures of muscle function have also been associated with treatment toxicity in older adults with cancer [33]. Sarcopenia in our cohort was associated with severe treatment toxicity among patients initiating an ARAT but not among those initiating chemotherapy. However, these results should be interpreted with caution due to the small sample, are hypothesis-generating and warrant further research.

Sarcopenia is associated with numerous adverse effects in older adults, including falls and fractures [34], hospitalizations [35], and may contribute to insulin resistance [36] and frailty [28], thereby increasing the risk of additional health-related adversities that may require immediate medical attention. Meta-analytic evidence of 2,832 older adults without cancer suggests that sarcopenia increases the risk of hospitalization [35]. In oncology, evidence on the impact of sarcopenia on health care utilization is limited with contradictory findings [37]. We found that participants initiating ARAT with sarcopenia had a significantly higher risk of visiting the ER compared with their non-sarcopenic counterparts. Nonetheless, a significant association between sarcopenia and the risk of visiting the ER was not observed in participants who were starting on chemotherapy. The lack of the association in the chemotherapy group may be a result of the small sample or other reasons that warrant further research.

Previous studies in men with mCRPC have found that sarcopenia measures are associated with disease progression [9, 10]. Specifically, Pak and colleagues found that patients with low muscle mass had shorter PSA progression-free survival and radiographic progression-free survival than patients with higher muscle mass based on median values [9]. Similarly, evidence from another study found that low skeletal muscle volume was a significant prognosticator of tumor progression. Our study corroborates previous evidence on the associations between sarcopenia and disease progression in men with mCRPC. Sarcopenic participants achieved PSA progression and radiographic progression earlier compared with their non-sarcopenic counterparts as indicated by the survival curves and the log rank test. In the adjusted analysis, sarcopenia was not a significant predictor of PSA progression possibly due to the issue of power. However, sarcopenia was a significant prognosticator of radiographic progression along with initiation of chemotherapy and higher creatinine levels. The exact mechanisms that precipitate the associations between sarcopenia and disease progression cannot be elucidated due to the nature of this study. However, in the presence of sarcopenia, several biological factors may favor cancer progression, such as increased systemic inflammation [38], immunosenescence [39], insulin resistance [36], or altered pharmacokinetics that may influence response to cancer treatment [40, 41].

Sarcopenia in our cohort was significantly associated with overall mortality in line with meta-analytic evidence in oncology [4]. Notably, participants without sarcopenia survived ~2 years longer compared with sarcopenic participants. Sarcopenia may increase the risk of all-cause mortality via numerous pathways that can either accelerate disease progression or decrease physiologic reserves that may limit mobility, expedite frailty and potentially disability. Another possible mechanism is that sarcopenia is associated with more rapid loss of fitness, limiting use of downstream life-prolonging treatments. A limitation of our study is that we did not examine these downstream treatments. In our cohort, initiating chemotherapy was associated with all study outcomes. Considering clinical sequencing of these drugs, this indicates that patients who are treated with chemotherapy have more advanced disease rather than chemotherapy is worse than ARAT.

Our findings are clinically relevant and highlight the importance of assessing and managing sarcopenia in clinical practice and warrant further research. Given the prognostic value of sarcopenia, an important question is how clinicians can efficiently and reliably assess sarcopenia in clinical practice. CT scan-based measures may be impractical to use in daily clinical practice. Alternatively, clinicians may choose to use simple and well-validated tests, such as grip strength and gait speed to assess sarcopenia in the clinical setting, adhering to the SDOC definition of sarcopenia [19, 31]. However, muscle-related parameters are central to the definition of sarcopenia and should also be assessed. Efforts to mitigate or reverse sarcopenia are of utmost importance for improving patient quality of life and potential clinically-relevant outcomes. In a systematic review of cancer survivors by Cao and colleagues, six of the seven exercise trials found that exercise increased skeletal muscle mass from baseline and compared to controls [42]. Additionally, meta-analytic evidence of 11 randomized controlled trials demonstrated that exercise, specifically resistance training improved muscle function and lean body mass in cancer survivors [43]. In addition to exercise, adequate protein ingestion is essential for increasing muscle protein synthesis [44] and mitigating anabolic resistance [45] in older patients. Therefore, clinicians are strongly encouraged to refer patients with sarcopenia and those at risk to qualified exercise professionals (Exercise Physiologist or Registered Kinesiologist) and dietitians.

The greatest strength of this study is the comprehensive definition of sarcopenia which involved measures of muscle mass and function. Additionally, assessment of muscle mass and quality through CT scans was obtained from the average of 5 slices which may provide a more accurate representation of SMI and SMD at the 3^rd^ lumbar vertebra. The greatest limitation of this study is the small sample, which may have diminished the impact of sarcopenia on several study outcomes, as evidenced by wide confidence intervals around some estimates. The limited sample also precluded an analysis by ARAT type to examine potential differences of the associations between sarcopenia and study outcomes. Additionally, albumin and lactate dehydrogenase were not included in multivariable analyses due to substantial data missingness that would further reduce our sample. Another limitation is the lack of exercise or physical activity data during the study period which may have altered some sarcopenia measures, particularly muscle strength and slowness. An inherent limitation from a clinical standpoint is the use of CT scans to quantify skeletal muscle mass and density. As described in the methods, skeletal muscle mass and density were assessed using a fully-automated algorithm, and any incorrect algorithm-derived segmentations were corrected manually using a commercially available, specialized software. However, these processes may be impractical in clinical practice due to time constraints and/or lack of expertise. These limitations urge the importance of developing reliable algorithms that can automatically quantify muscle mass and density without the need of manual editing through a medical imaging software. Lastly, another limitation is the lack of reasons for ER use.

## Conclusion

Sarcopenia defined by low muscle mass or quality in addition to low muscle strength and slowness predicts radiographic progression and overall mortality in men with mCRPC initiating chemotherapy or ARAT. Additionally, sarcopenia may predict severe treatment toxicity and ER visits prior to ARAT initiation for mCRPC. Larger studies are warranted to elucidate the impact of sarcopenia on clinical outcomes in men with mCRPC.

## Supporting information

S1 File (DOCX)
