# Correction: The impact of sarcopenia on clinical outcomes in men with metastatic castrate-resistant prostate cancer

**DOI:** 10.1371/journal.pone.0314372

**Published:** 2024-11-19

**Authors:** Efthymios Papadopoulos, Andy Kin On Wong, Sharon Hiu Ching Law, Lindsey Ze Jing Zhang, Henriette Breunis, Urban Emmenegger, Shabbir M. H. Alibhai

The mean gait speed listed in Table 1 is incorrect. Participants with sarcopenia had a mean gait speed of 0.6m/s (SD: 0.1), whereas the mean gait speed of participants with no sarcopenia was 1.0m/s (SD: 0.2). Please see the correct Table 1 here.

**Table 1:** Baseline characteristics of study participants.

Characteristic	All participants n = 110	Sarcopenia^a^ n = 30	No sarcopenia n = 80	*p*	Missing n (%)
*Sociodemographic characteristics*					
Age (years), mean (SD)	74.6 (7.1)	75.7 (7.6)	74.1 (6.8)	0.30	0 (0)
BMI (kg/m^2^), mean (SD)	27.5 (4.7)	26.5 (4.9)	27.9 (4.6)	0.18	0 (0)
Race					0 (0)
Black	13 (11.8)	3 (10.0)	10 (12.5)	0.76	
South Asian	7 (6.4)	3 (10.0)	4 (5.0)		
Southeast Asian	8 (7.3)	3 (10.0)	5 (6.3)		
Unknown	1 (0.9)	0 (0)	1 (1.3)		
White	81 (73.6)	21 (70.0)	60 (75.0)		
Education, n (%)					0 (0)
Completed college/university or graduate degree	58 (52.7)	15 (50.0)	43 (53.8)	0.72	
*Clinical characteristics*					
Gleason score ≥ 8, n (%)	46 (48.9)	11 (45.8)	35 (50.0)	0.72	16 (14.5)
ADT duration (years), mean (SD)	6.2 (4.9)	6.4 (4.6)	6.1 (5.0)	0.73	0 (0)
Treatment type, n (%)				0.002	0 (0)
ARAT	63 (57.3)	10 (33.3)	53 (66.3)		
Chemotherapy	47 (42.7)	20 (66.7)	27 (33.8)		
Bone metastasis, n (%)	80 (72.7)	20 (66.7)	60 (75.0)	0.38	0 (0)
PSA most proximal to baseline, median (IQR), nmol/L	26.0 (8.9–76.1)	78.0 (76.0)	146.3 (806.9)	0.65	2 (1.8)
*Comorbidities*					
MoCA, mean (SD)	24.6 (3.6)	24.9 (3.5)	24.4 (3.7)	0.59	1 (0.9)
Dependence in one or more IADLs, n (%)	41 (37.3)	21 (70.0)	20 (25.3)	<0.001	1 (0.9)
VES-13 ≥3, n (%)	30 (27.3)	17 (56.7)	13 (16.3)	<0.001	0 (0)
Arthritis, n (%)	53 (48.2)	15 (50.0)	38 (47.5)	0.81	0 (0)
Congestive heart failure, n (%)	5 (4.5)	1 (1.3)	4 (13.3)	0.007	0 (0)
Diabetes, n (%)	22 (20.0)	8 (26.7)	14 (17.5)	0.28	0 (0)
Hyperlipidemia, n (%)	46 (41.8)	14 (46.7)	32 (40.0)	0.53	0 (0)
Hypertension, n (%)	60 (54.5)	21 (70.0)	39 (48.8)	0.046	0 (0)
Osteoporosis, n (%)	7 (6.4)	3 (10.0)	4 (5.0)	0.34	0 (0)
*Blood markers*					
Albumin (g/L), mean (SD) units	40.1 (3.5)	37.9 (3.6)	41.0 (3.1)	<0.001	15 (13.6)
Alkaline phosphatase (u/l), median, (IQR)	96.0 (74.0–143.3)	194.9 (184.2)	130.0 (150.1)	0.10	12 (10.9)
Lactate dehydrogenase (u/l), mean (SD)	260.9 (99.2)	308.14 (132.61)	240.5 (73.0)	0.016	17 (15.5)
Hemoglobin (g/L), mean (SD)	123.5 (15.9)	112.34 (17.0)	128.0 (12.9)	<0.001	10 (9.1)
Creatinine (IU/L), mean (SD)	99.7 (50.2)	111.1 (52.19)	94.9 (48.9)	0.14	10 (9.1)
*Sarcopenia-related measures*					
Grip strength (kg), mean (SD)	31.8 (7.8)	25.6 (4.7)	34.1 (7.4)	<0.001	0 (0)
Gait speed (m/s), mean (SD)	0.93 (0.2)	0.6 (0.1)	1.0 (0.2)	<0.001	0 (0)
Grip strength, n (%)					
<35.5kg	74 (67.3)	30 (100.0)	44 (55.0)	<0.001	0 (0)
Gait speed, n (%)					0 (0)
<0.8m/s	36 (32.7)	30 (100.0)	6 (7.5)	<0.001	
Days from CT scan to treatment initiation, mean (SD)	46.2 (41.2)	43.6 (37.8)	47.1 (42.6)	0.69	0 (0)
Skeletal muscle index, cm^2^/m^2^, mean (SD)	42.9 (7.4)	39.9 (6.5)	44.0 (7.3)	0.008	0 (0)
Muscle density (HU), mean (SD)	27.2 (9.8)	25.2 (9.3)	27.9 (9.9)	0.19	0 (0)
Low skeletal muscle index, n (%)	91 (82.7)	28 (93.3)	63 (78.8)	0.072	0 (0)
Low skeletal muscle density, n (%)	86 (78.2)	28 (93.3)	58 (72.5)	0.018	0 (0)

^a^Sarcopenia was defined as the presence of low muscle strength (grip strength <35.5kg), low gait speed (walking speed <0.8m/s), and low muscle quantity or quality.

ADT = androgen deprivation therapy; ARAT = androgen receptor-axis targeted therapy; BMI = body mass index; IADLs = instrumental activities of daily living; MoCA = Montreal cognitive assessment; PSA = prostate-specific antigen; VES-13 = Vulnerable Elders Survey-13
