# Supplementary material for: The impact of sarcopenia on clinical outcomes in men with metastatic castrate-resistant prostate cancer
Source: PLoS One. 2023 Jun 1;18(6):e0286381. doi: 10.1371/journal.pone.0286381 (PMC10234556; doi:10.1371/journal.pone.0286381)
Supplement: S1 File — (DOCX) [file pone.0286381.s001.docx]

Supplemental Tables

**S1 Table.** Incidence of grade 3+ toxicity, PSA progression, radiographic progression, and overall mortality

|  | Grade 3+ toxicity | PSA progression | Radiographic  progression | Overall mortality | ER visits |
| --- | --- | --- | --- | --- | --- |
| n (%) | 42 (38.2) | 66 (60.0) | 50 (45.5) | 64 (64.6)^a^ | 45 (40.9) |
| Missing data  n (%) | 4 (3.6)* | 0 (0) | 0 (0) | 0 (0) | 7 (6.4) |

*n=4 discontinued treatment prior to collection of toxicity data

^a^n=11 participants were re-enrolled in the study after switching treatment. For overall mortality, participants were included once, from the time of primary active treatment until death or loss to follow up.

**S2 Table.** Univariate and multivariable logistic regression of the impact of sarcopenia on severe treatment toxicity

| Variable | Multivariable  OR (95%CIs)  (n=106)  MODEL WITH NO INTERACTION | P | Multivariable  OR (95%CIs)  (n=106)  MODEL WITH INTERACTION | P |
| --- | --- | --- | --- | --- |
| Age per decade | 1.31 (0.70-2.44) | 0.39 | 1.25 (0.67-2.36) | 0.48 |
| Treatment type |  |  |  |  |
| Chemotherapy | 3.57 (1.44-8.89) | **0.006** | 5.95 (2.01-17.63) | **0.001** |
| ARAT | ref. |  | ref. |  |
| Sarcopenia |  |  |  |  |
| Yes | 1.75 (0.65-4.72) | 0.27 | 5.89 (1.12-30.93) | **0.036** |
| No | ref. |  | ref. |  |
| Sarcopenia * Treatment Type |  |  | 0.15 (0.02-1.16) | 0.069 |
| Anemia |  |  |  |  |
| Yes | 3.05 (0.89-10.41) | 0.075 | 2.96 (0.85-10.35) | 0.089 |
| No |  |  |  |  |

ARAT= androgen receptor-axis targeted therapy

MODEL WITH NO INTERACTION

Hosmer-Lemeshow test: 12.18, p=0.14

C-stat: 0.75

MODEL WITH INTERACTION

Hosmer-Lemeshow test: 4.77, p=0.68

C-stat: 0.75

**S3 Table.** Univariate and multivariable Cox regression of the impact of sarcopenia on the time to the first emergency room visit

| Variable | Multivariable  OR (95%CIs)  (n=94)  MODEL WITH NO INTERACTION | P | Multivariable  OR (95%CIs)  (n=94)  MODEL WITH INTERACTION | P |
| --- | --- | --- | --- | --- |
| Age per decade | 1.91 (1.16-3.15) | **0.011** | 1.86 (1.11-3.11) | **0.019** |
| Treatment type |  |  |  |  |
| Chemotherapy | 9.84 (3.86-25.10) | **<0.001** | 17.33 (5.56-53.95) | **<0.001** |
| ARAT | ref. |  | ref. |  |
| Sarcopenia |  |  |  |  |
| Yes | 1.63 (0.77-3.42) | 0.19 | 4.37 (1.30-14.68) | **0.017** |
| No | ref. |  | ref. |  |
| Sarcopenia * Treatment Type |  |  | 0.25 (0.63-1.02) | 0.053 |
| Anemia |  |  |  |  |
| Yes | 1.27 (0.41-3.93) | 0.67 | 1.24 (0.40-3.79) | 0.70 |
| No | ref. |  | ref. |  |
| Hypertension |  |  |  |  |
| Yes | 1.42 (0.72-2.82) | 0.31 | 1.41 (0.70-2.82) | 0.33 |
| No | ref. |  | ref. |  |
| Hemoglobin per 10g/L | 0.88 (0.69-1.12) | 0.29 | 0.91 (0.71-1.15) | 0.44 |

ARAT= androgen receptor-axis targeted therapy
